# Supplementary material for: Pooled‐matrix protein interaction screens using Barcode Fusion Genetics
Source: Mol Syst Biol. 2016 Apr 23;12(4):863. doi: 10.15252/msb.20156660 (PMC4848762; doi:10.15252/msb.20156660)
Supplement: Supplementary file 1 — Appendix [file MSB-12-863-s001.pdf]

Appendix for

Pooled-matrix protein interaction screens using Barcode Fusion Genetics

Nozomu Yachie[1–5*,16], Evangelia Petsalaki[1,2,*], Joseph C. Mellor[1,2], Jochen Weile[1,2,6], Yves Jacob[7], Marta Verby[1,2], Sedide B. Ozturk[1,2], Siyang Li[1,2], Atina G. Cote[1,2], Roberto Mosca[8], Jennifer J. Knapp[1,2], Minjeong Ko[1,2], Analyn Yu[1,2], Marinella Gebbia[1,2], Nidhi Sahni[9,10], Song Yi[9,10], Tanya Tyagi[1,2], Dayag Sheykhkarimli[1,2,6], Jonathan F. Roth[1,2,6], Cassandra Wong[1,2], Louai Musa[1,2], Jamie Snider[1], Yi-Chun Liu[1], Haiyuan Yu[11], Pascal Braun[9,10,12], Igor Stagljar[1,6], Tong Hao[9,10], Michael A. Calderwood[9,10], Laurence Pelletier[2,6], Patrick Aloy[8,13], David E. Hill[9,10], Marc Vidal[9,10] & Frederick P. Roth[1,2,6,9,14,15,16]

[1]Donnelly Centre, University of Toronto, Toronto, Ontario, Canada. [2]Lunenfeld-Tanenbaum Research Institute, Mt. Sinai Hospital, Toronto, Ontario, Canada. [3]Synthetic Biology Division, Research Center for Advanced Science and Technology, the University of Tokyo, Tokyo, Japan. [4]Institute for Advanced Bioscience, Keio University, Tsuruoka, Yamagata, Japan. [5]PRESTO, Japan Science and Technology Agency (JST), Tokyo, Japan. [6]Department of Molecular Genetics, University of Toronto, Toronto, Ontario, Canada. [7]Département de Virologie, Unité de Génétique Moléculaire des Virus à ARN, Institut Pasteur, Paris, France. [8]Joint IRB-BSC Program in Computational Biology, Institute for Research in Biomedicine (IRB Barcelona), Barcelona, Spain. [9]Center for Cancer Systems Biology (CCSB) and Department of Cancer Biology, Dana-Farber Cancer Institute, Boston, Massachusetts, USA. [10]Department of Genetics, Harvard Medical School, Boston, Massachusetts, USA. [11]Weill Institute for Cell and Molecular Biology, Cornell University, Ithaca, New York, USA. [12]Technische Universität München, Wissenschaftszentrum Weihenstephan, Department of Plant Systems Biology, Freising, Germany. [13]Institució Catalana de Recerca i Estudis Avançats (ICREA), Barcelona, Spain. [14]Canadian Institute for Advanced Research, Toronto, ON. [15]Department of Computer Science, University of Toronto, Toronto, Ontario, Canada.
*These authors contributed equally to this work.

[16]Correspondence should be addressed to N.Y. (yachie@synbiol.rcast.u-tokyo.ac.jp) or F.P.R. (fritz.roth@utoronto.ca).

Table of Contents

**Note S1**	Proof-of-principle demonstrations of BFG-Y2H
**Note S2**	Row-Column-Plate (RCP)-PCR
**Note S3**	Theoretical estimation of screening complexities
**Note S4**	Processing BFG-Y2H sequencing reads
**Figure S1**	Monte-Carlo simulation of the BFG-Y2H process
**Figure S2**	Performance of the huCENT screen with various interaction scoring methods
**Figure S3**	Top 100 protein pairs scored in the CCC screen
**Figure S4**	Cost comparison of primary screening by state-of-the-art Y2H versus *en masse* recombinational cloning (EMRC)-based BFG-Y2H

Note S1. Proof-of-principle demonstrations of BFG-Y2H

Theory on serial recombination events required for barcode swapping

Cre-mediated recombination events vary in order, producing alternative pathways of resulting fused barcode products. Two pathways theoretically produce the majority of barcode fusion products (**Figure EV2a**). The first pathway starts with the fusion of query bait and prey plasmid molecules via *loxP* sites, producing an intermediate dimeric plasmid product containing a fusion event between BC1 barcodes ('BC1-BC1 fusion'). A subsequent intramolecular recombination between the two *lox2272* sites splits the dimerized plasmid into a bait plasmid variant containing a fusion event between BC2 barcodes ('BC2-BC2' fusion) and an prey plasmid variant containing the BC1-BC1 fusion. The second pathway starts with the fusion of bait and prey plasmids via the *lox2272* sites first, producing another type of dimeric plasmid containing a BC2-BC2 fusion, which can subsequently split into bait and prey plasmids containing BC2-BC2 and BC1-BC1 fusions, respectively, by *loxP* recombination. By either pathway, two barcode fusions are yielded via the physical swapping of bait-BC1 and prey-BC2 cassettes.

The Cre-mediated *loxP* and *lox2272* recombination reactions are reversible. In heterogeneous cell culture, populations of intermediate dimer products are likely present at lower concentrations than plasmid monomers, given the greater efficiency expected for intramolecular recombination events. Also, plasmid loss may occur more frequently for dimeric plasmids, as plasmids with multiple centromeres may fragment when kinetochores attach centromeres of same sister chromatid to opposite spindle poles (Neumann et al, 2012).

Demonstration of serial Cre-mediated recombination and barcode swapping

We prepared three diploid strains each carrying barcoded bait and prey plasmids: two diploid strains with the 'toolkit' RY-strain background and one diploid strain with the common Y2H Y-strain background. Each strain was separately cultured with doxycycline overnight to induce the Cre-mediated barcode fusion and then split into two cultures: one culture was treated with cycloheximide (CHX) to select for loss of the *CYH2* gene encoded on the prey plasmid, while the other culture was not. We lysed yeast cells from the CHX-treated and untreated cultures, and performed direct PCRs (using yeast lysate as the immediate source of template) for the bait and prey non-recombined BC1-BC2 barcode regions and the BC1-BC1 and BC2-BC2 fused barcode regions. The PCR results demonstrated the presence of BC2-BC2 fusion after the CHX treatment (**Figure EV2b**). Both plasmid dimers (chimeras of bait and prey plasmids) should encode the *CYH2* gene, so that after CHX-treatment only bait monomers should remain. Thus, the presence of BC2-BC2 fusion after CHX treatment supported the idea that Cre-mediated recombination split dimeric plasmids into monomeric plasmids containing fused-barcodes.

Barcode fusion happens only within diploid BFG-Y2H toolkit cells

To demonstrate proof of principle for *in vivo* Cre-mediated barcode fusion, we prepared barcoded bait plasmids Bt1 (encoding LCP) and Bt2 (FKBP3) and prey plasmids Py1 (NCK1) and Py2 (NQO2) by Gateway LR cloning, and generated bait and prey trial strains RY-Bt1, RY-Bt2, RY-Py1 and RY-Py2 with the 'toolkit' background strains RY1030 for baits and RY1010 for preys, and Y-Bt1 with the Y8930 background and Y-Py1 with the Y8800 background.

All four bait-prey pairs were mated to create diploid cells with the toolkit strain background. We also generated one diploid strain with the Y-strain background from the haploid strains Y-Bt1 and Y-Py1. We cultured the five diploid strains and haploid RY-Bt1 and RY-Py1 strains separately in appropriate selection media (SC–Leu–Trp+Ade for diploid strains, SC–Leu+Ade and SC–Trp+Ade for bait and prey haploid strains, respectively) and treated the cell cultures with doxycycline overnight. Subsequently, all of the seven cell samples (four RY diploid, two RY haploid and one Y diploid) were lysed and aliquots of RY-Bt1 and RY-Py1 haploid cell lysates were mixed in 1:1 volume. For each of the eight PCR template samples, we performed PCRs targeting the non-recombined BC1-BC2 barcode regions of the query bait and prey plasmids and the BC1-BC1 and BC2-BC2 fused barcodes (**Figure EV2c**).

We observed both non-recombined and fused-barcode products for all four of the toolkit diploid cell samples. However, fused barcode products were not observed for cells in the Y-strain background with bait and prey plasmids, nor were they seen for haploid cell samples for which the cells carried only bait or only prey plasmids. RY-Bt1 samples showed only the bait query non-recombined barcode product and RY-Py1 showed only the query prey barcode product. The mixture of RY-Bt1 and RY-Py1 lysates showed both of the bait and prey barcode products but not the fused barcode product, as expected given that bait and prey plasmids should never both be within the same cell. These results confirmed that barcode fusion is specific to plasmids within the same cell and not the result of PCR template switching, or Cre recombination between extracellular plasmids from lysed cells.

Fused barcodes represent abundance in mixed cell populations

To further assess the specificity of intracellular Cre-mediated barcode fusion, we generated a 1:1 mixture of the Bt1-Py1 diploid cells and the Bt2-Py2 diploid cells in the toolkit background, treated this mixture with doxycycline to induce barcode fusion, lysed cells, and separately amplified fused-barcode products of the BC1-BC1 and BC2-BC2 fusions. We cloned the resulting PCR products and sequenced 24 clones of BC1-BC1 fused barcodes and 24 clones of BC2-BC2 fused barcodes isolated by bacterial transformation and single colony isolation. The same experiment was also performed for the mixture of the Bt1-Py2 and Bt2-Py1 diploid cells and 23 clones were sequenced for BC1-BC1 fusion and 24 clones were sequenced for BC2-BC2 fusion.

Most of the sequenced fused barcodes corresponded to the expected Bt-Py combination (**Figure EV2d**; 94 out of 95 clones), indicating that the majority of the barcode fusions happened within the cell and that the Cre-mediated barcode fusion enables us to identify specific X-Y protein pairs from a mixed population. Only one clone of the BC2-BC2 fusion from the Bt1-Py2 and Bt2-Py1 mixture showed an unexpected fused barcodes (Bt1-Py1), implying that a small proportion of non-specific fused barcodes could be produced, presumably by non-specific extracellular barcode fusion after cell lysis or template switching between fused barcodes during PCR amplification.

Barcode fusion efficiency after BFG-Y2H

We performed rolling circle DNA amplification (RCA) and Illumina Nextera sequencing of the plasmid DNA pools extracted from yeast cells after the CENT BFG-Y2H +His and –His screens (**Figure EV5a**). Plasmid DNA pools extracted from yeast cell cultures were amplified by the φ29-based rolling circle amplification (RCA) using TempliPhi DNA Amplification Kits (GE Healthcare Life Sciences). The sequencing libraries were prepared from the amplified DNA using Nextera DNA Sample Prep Kit (Illumina) and analyzed by MiSeq (Illumina; 2×150-bp paired-end sequencing). We counted sequence reads having both upstream and downstream 7-bp flanking sequences of *loxP* or *lox2272* sites (**Figure 4e** and **Figure EV5f**). We found that an average of 17.8% and 15.8% of the *loxP* sites and *lox2272* sites, respectively, were recombined in the +His condition, and 27.3% (*loxP*) and 23.7% (*lox2272*) were recombined in the –His condition.

Note S2. Row-Column-Plate (RCP)-PCR

PCR design and primers

To determine the quality of clonal barcode-carrier bait and prey plasmids and to identify the specific barcode sequences within the arrayed collection, we performed Row-Column-Plate PCR (RCP-PCR; **Figure EV3b**) in 384-well plates. Due to the limitations in read length of Illumina MiSeq sequencing at the time (2×150-bp paired-end, Illumina MiSeq kit version 1), we carried out different types of RCP-PCR on bait and prey barcode-carrier plasmids: 'bait-BC' and 'prey-BC' RCP-PCRs were designed to identify barcodes within each plate well; and 'bait-lox' and 'prey-lox' RCP-PCRs were designed to validate the sequence identity of *loxP* and *lox2272* sites for each plate well. For each of the RCP-PCR variants, we prepared 16 index-tagged row primers and 24 index-tagged column primers, and 12 plate primers (6 forward and 6 reverse) to encode the identity of as many as 36 384-well plates (**Appendix Table S5**) such that all identities could be decoded in a single next-generation sequencing run. The detailed PCR design was as follows.

Bait-BC and bait-lox RCP-PCRs

```
          >>>>>>>>>>>>>>>>>>>>                                  >>>>>>>>>>>>>>>
CTCACTAAAGGGAACAAAAGCTGGGTACCGATAACTTCGTATAATGTATGCTATACGAAGTTATCCATACGAGCACATT
                              [------------ loxP’* ------------][----- DB-U1 --
>>>>>
ACGGGNNNNNNNNNNNNNNNNNNNNNNNNNCTAACTCGCATACCTCTGATAACATAACTTCGTATAGGATACTTTATAC
----][------ BAIT-BC1  ------][------- DB-U2 -------][----------- lox2272 -----
        <<<<<<<<<<<<<<<<<<<<<<<<<<
GAAGTTATTGTCAGCACTCTGTCAAAATAGATCGGAAATCGATAGGTGCGTGTGAAGGNNNNNNNNNNNNNNNNNNNNN
-------][---------- LINKER ----------][----- DB-D1 ------][------ BAIT-BC2  ---
    <<<<<<<<<<<<<<<<<<<
NNNNCCTCAGTCGCTCAGTCAAGCCCCCTCGAGATCCGGGATCGAAGAAATGA
---][----- DB-D2 -----]
```

Prey-BC and prey-lox RCP-PCRs

```
                              >>>>>>>>>>>>>>>>>>>>
GAAGGCTTTAATTTGCAAGCTCGGGATCCGCCCTTAGAACCGAGAGTGTGNNNNNNNNNNNNNNNNNNNNNNNNNCACT
                              [----- AD-U1 ------][------ PREY-BC1  ------][---
                     >>>>>>>>>>>>>>>>>>>>>>>>
CCGTTCGTCACTCAATAATGTCAGCACTCTGTCAAAATAGATCGGAAAATAACTTCGTATAATGTATGCTATACGAAGT
--- AD-U2 -------][---------- LINKER ----------][------------ loxP’* ----------
                                               <<<<<<<<<<<<<<<<<<<
TATCTCCAGGGTTAGGCAGATGNNNNNNNNNNNNNNNNNNNNNNNNNCAATCGCACTATCCCGCTGATAACTTCGTATA
--][----- AD-D1 -----][------ PREY-BC2  ------][----- AD-D1 -----][------------
                            <<<<<<<<<<<<<<<<<<<<<<
GGATACTTTATACGAAGTTATCCCCCTCGAGATCCGGGATCGAAGAAATGA
lox2272 ------------]
```

>>>>> <<<<< Row and column primer 'landing sites' for bait-BC and prey-BC RCP-PCR (blue)
>>>>> <<<<< Row and column primer 'landing sites' for bait-lox and prey-lox RCP-PCR (red)
**loxP'* is reverse complement of *loxP*

RCP-PCR procedure

For Row-Column (RC)-PCR, we used a high-capacity thermal cycler (Hydrocycler 16, KBioscience) capable of thermally cycling up to 16 384-well plates at once, with the following protocol:

RC-PCR setup (10-uL rxn/well)

5× Phusion HF buffer	2 µl
25 mM dNTPs	0.08 µl
Phusion DNA polymerase	0.1 µl
ddH2O	1.82 µl
2 uM Row primer	1 µl
2 uM Column primer	1 µl
~20-fold dilution of *E. coli* overnight culture	4 µl
Total volume	10 µl

PCR program

1	95 °C for 10 min
2–5	95 °C for 10 sec, 63 °C for 10 sec, 72 °C for 15 sec (30 cycles)
6	72 °C for 5 min
7	4 °C forever

For each of the different RCP-PCR variants, RC-PCR samples were pooled and purified separately for each plate, and gel band of the target size was selected on 4% agarose gel and subjected to Plate-PCR:

Plate-PCR setup (40-uL rxn)

2× Phusion High-Fidelity PCR Master Mix	20 µl
10 uM Forward plate primer	1 µl
10 uM Reverse plate primer	1 µl
~1 ng μl^{-1} size-selected RC-PCR product	5 µl
ddH2O	13 µl
Total volume	40 µl

PCR program

1	98 °C for 30 sec
2–5	98 °C for 10 sec, 60 °C for 10 sec, 72 °C for 1 min (15 cycles)
6	72 °C for 5 min
7	4 °C forever

Each Plate-PCR sample was purified, and the gel band of the target size was selected on 4% agarose gel, quantified by qPCR, multiplexed with other libraries, and sequenced by Illumina MiSeq (2×150-bp paired-end sequencing).

Data analysis of sequencing reads

For each paired-end read, sequences were analyzed to extract the row-column-plate index combinations (identifying the physical plate-well coordinates), sequence regions identifying the RCP-PCR variant type, and other sequences in the barcode region: each 'bait-BC' read

contained the bait-BC1, *lox2272* site and bait-BC2; each ‘bait-lox’ read contained the *loxP* site, bait-BC1 and *lox2272* site; each ‘prey-BC’ read contained the prey-BC1, *loxP* site and prey-BC2; and each ‘prey-lox’ read contained the *loxP* site, prey-BC2 and *lox2272* site. For each well, bait-BC or prey-BC reads were clustered into groups according to BC1 and BC2 sequences, and then bait-lox and prey-lox reads were assigned to corresponding bait-BC and prey-BC groups according to bait-BC1 and prey-BC2, respectively. Each read group was used to assess quality of the *loxP* and *lox2272* sites and the PCR priming sites flanking the barcodes. Plate wells having a single pair of BC1 and BC2 with acceptable sequence elements were identified. Analysis scripts are available on request.

Note S3. Theoretical estimation of screening complexities

We developed a Monte-Carlo simulation of the BFG-Y2H experiment to determine the appropriate scale of each stage of the experiment, so that a sufficient average number of cells per strain were used and a sufficient number of reads per fused barcode were obtained to maintain high sensitivity and reproducibility (**Appendix Figure S1**). The experimental scale used for the human centrosomal BFG-Y2H screen is followed by a brief description of experimental procedures and average sample complexities simulated for a 400×400 screen (400 bait and 400 prey strains, n = 1,000). Variation of barcode copy numbers in Y2H-selective and non-Y2H-selective conditions derived from the simulation matched well with those observed experimentally (**Appendix Figure S1** and **Figure EV4**). Program scripts are available on request.

Modeling choices and parameters used for simulating the BFG-Y2H process

P1. Strain abundances in the initial haploid pools follow a log-normal distribution with CV (Coefficient of Variation) = 30%

P2. 1 OD_{600} nm unit for haploid yeast is 3×10^7 cells ml^{-1}

P3. X-Y pair-dependent mating efficiencies* during the mating process, follow a log-normal distribution with CV = 50%

P4. Overall yeast mating efficiency* of Y2H is 1% (a conservatively low estimate) (Bickle et al, 2006)

P5. X-Y-dependent growth** in liquid media follows a log-normal distribution with CV = 50%

P6. 1 OD_{600} nm unit for diploid yeast is 1×10^7 cells ml^{-1}

P7. X-dependent growths in the Y2H selective media (e.g. autoactivities) follow a log-normal distribution with CV = 100%

P8. X-Y-dependent growth** in the Y2H selective media follow a log-normal distribution with CV = 10,000%

P9. Y2H positive rate (ratio of number of colony forming units under Y2H selective condition to the equivalent number in non-selective media) is 0.1% (overestimation) (Rolland et al, 2014)

P10. X-Y-dependent growth** in non-selective media follows a log-normal distribution with CV = 10%

P11. Yeast DNA mini-prep yield from 3×10^7 diploid yeast cells is 30 ng

P12. Y2H plasmid size is 10 kbp

P13. Double-stranded DNA is 660 g bp^{-1} $mole^{-1}$

P14. Fraction of Y2H plasmids in yeast DNA miniprep product is 6% of total mass (**Figure EV5**)

P15. Barcode fusion efficiency is 20% (**Appendix Note S1**)

P16. X-Y-dependent PCR yield follows a log-normal distribution with CV = 50%
P17. Number of sequencing reads obtained from each screen is 10,000,000

*Ratio in number of colony forming units (CFUs) on diploid selective media to that on rich (non-selective media)
**Relative abundance of cells from before cell culture to after cell culture

Step I. Preparation of haploid pools

Experimental procedure
Bait strains and prey strains were respectively pooled in 1-liter flasks. For each pool, cell concentration was adjusted so a 100-fold dilution yields an OD_{600} nm = 1.0. 10 ml of bait pool and 10 ml of prey pool were then mixed for yeast mating.

Simulation results
In each simulation run, each haploid pool was generated by mixing 400 strains with a heterogeneity given by the modeling parameter **P1**. From each pool, 10 ml cells at 1.0 OD_{600} nm (3×10^{10} cells given by **P2**) were taken from each of two opposite mating-type pools and combined into a single yeast mating pool. Each haploid pool sample provided $\geq 10^7$ cells for each of the haploid strains with CV of 30.0% for bait and CV = 29.9% for prey (**Appendix Figure S1**).

Step II. Yeast mating

Experimental procedure
The mating pool was incubated overnight at room temperature. After the mating, cells were washed and resuspended in 500 ml of diploid selection media at 1.0 OD_{600} nm in a 4-liter flask.

Simulation results
In the simulated mating sample pool from the **Step I**, bait and prey cells had X-Y pair-dependent mating efficiencies given by the modeling parameter **P3**. After the mating, 500 ml of the mating sample at 1.0 OD_{600} nm (1.5×10^{10} haploid cells given by **P1**) was carried into the next step. This sample (1.5×10^{10} diploid cells given by **P4**) yielded an average of ≥100 diploid cells for each strain within the tested space of X-Y pairs with CV = 69.7% (**Appendix Figure S1**).

Step III. Diploid enrichment and cell spreading

Experimental procedure
To enrich for diploid cells, the post-mating cell culture was incubated at 30 °C until the OD_{600} nm reached ~5.0. The concentration of the diploid sample was adjusted in a 50-ml Falcon tube so a 50-fold dilution yielded OD_{600} = 1.0. Then 200 µl of the sample was spread on each selection plate (150-mm Petri dishes).

Simulation results
In the simulated sample from **Step II**, diploid cells were enriched with X-Y pair specific growth effects given by the modeling parameter **P5**. The concentration of diploid cell sample was adjusted to 5×10^{10} cells/ml (given by **P6**) and 200 µl of the adjusted sample (1×10^{10} cells) was spread on each selection plate. To each plate, ≥10 cells were queried for each X-Y pair within the entire X-Y pair space with CV = 92.6% (**Appendix Figure S1**).

Step IV. Y2H selection

Experimental procedure
The Y2H selective plates and non-selective plates were incubated for two days at 30°C. Cells were then scraped and pooled.

Simulation results
In the colony-forming simulation of the Y2H-selective plates from the **Step III**, relative colony size formed from each cell was derived by a product of X-dependent growth (e.g. auto-activities) given by the modeling parameter **P7** and X-Y pair-dependent growth given by **P8**. X-Y protein pairs simulated to have top 0.1% (given by **P9**) X-Y pair-dependent growth in the Y2H selective condition were assigned to have 'true interactions'. Colonies were also formed on the non-selective plates given by **P10**. After the selections, CVs in distribution of X-Y diploid population size were 4228.0% and 96.6% in Y2H selective and non-selective conditions, respectively (**Appendix Figure S1**).

Step V. Doxycycline-treatment and yeast plasmid extraction

Experimental procedure
Each sample was diluted to adjust its concentration to 1.0 OD_{600} nm in 5 ml volume and incubated overnight at 30 °C with doxycycline for the Cre-mediated barcode fusion.

Plasmids were then extracted from 1 ml cell samples at 3.0 OD_{600} nm for each screening condition.

Simulation results

Each simulated sample obtained from the simulation **Step IV** was treated overnight with doxycycline with X-Y dependent growth effects given by the modeling parameter **P5**, and 30 ng of DNA was extracted from 3×10^8 cells (given by **P6**). Each DNA sample conferred 1.64×10^7 plasmid molecules with barcode fusion for each of BC1-BC1 and BC2-BC2 fusions (given by the modeling parameters **P11–15**). The Y2H selective condition sample contained ≥100 BC1-BC1 and ≥100 BC2-BC2 fusion plasmids for each interacting X-Y pair defined in the **Step IV** with CV = 4550.6% (**Appendix Figure S1**). The non-selective condition sample contained ≥1 BC1-BC1 fusion and ≥1 BC2-BC2 fusion plasmids for each of the X-Y pairs in the tested space (≥10 plasmids and ≥100 plasmids for 97.2% and 32.5% of the tested space) with CV = 119.6% (**Appendix Figure S1**).

Step VI. Sequencing of barcode loci

Experimental procedure

Sequencing libraries for the fused barcodes were prepared by PCR with primers having Illumina sequencing adapters with sample-multiplexing indices. Sequencing was performed by 2×150 MiSeq, NextSeq 500 or HiSeq 2500 runs using the manufacturer's instructions.

Simulation results

To prepare the sequencing library, each simulated DNA sample from **Step V** was subjected to PCR and fused barcodes were amplified with X-Y fused-barcode-dependent PCR biases given by the modeling parameter **P16**. A total of 10 million reads (given by **P17**) were simulated for each sample. From each sequencing run, ≥100 BC1-BC1 and ≥100 BC2-BC2 reads were obtained for each interacting X-Y pair defined in the **Step IV** with CV = 4860.2%, and ≥1 BC1-BC1 reads and ≥1 BC2-BC2 reads for 99.6% of the tested X-Y pair space (≥10 reads and ≥100 reads each for 86.7% and 16.3% of the tested X-Y pair space, respectively) with CV = 144.0% (**Appendix Figure S1**).

Note S4. Processing BFG-Y2H sequencing reads

The in-yeast assembly-based BFG-Y2H screens

In in-yeast assembly-based BFG-Y2H screen, interaction signal *s'* was calculated for each of BC1-BC1 and BC2-BC2 barcode fusions as follows:

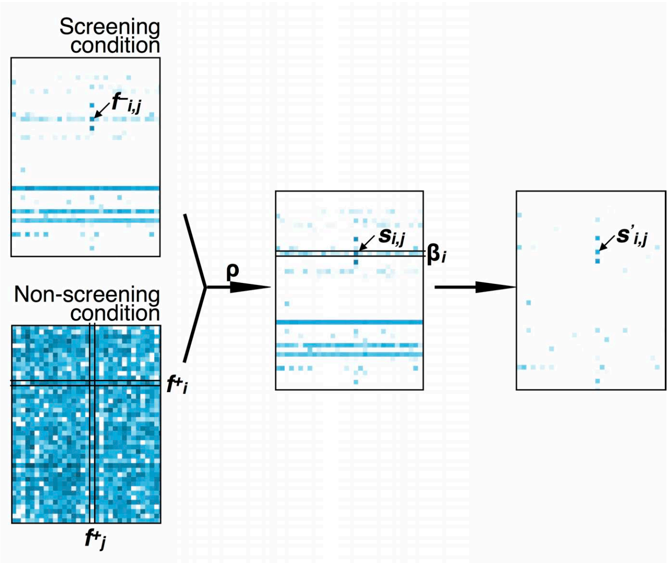

Let $c^-_{i,j}$ and $c^+_{i,j}$ be read counts of fused barcodes of strain pair X_i-Y_j in the Y2H selective (c^-) and non-selective (c^+) conditions, respectively. A normalized diploid population estimate $f^-_{i,j}$ was obtained according to ($c^-_{i,j}$ + α) divided by the total read count (the constant α was set to 1.0 throughout this study). Due to the limited read depth and the high sample complexity for every strain pair X_i-Y_j in the non-selective condition, the frequency $f^{+,\sim}_{i,j}$ of each diploid cell type under non-selective conditions was derived as the product of $f^+_i \times f^+_j$, where f^+_i and f^+_j are sums of $f^+_{i,j}$ corresponding to X_i and Y_j, respectively. (In other words, we estimated the frequency of each strain type under non-selective conditions as the product of frequency totals for the corresponding row and column) The enrichment signal for each fused barcode ($s_{i,j}$) was then calculated as $f^-_{i,j} / f^{+,\sim}_{i,j}$.

In a BFG-Y2H matrix with quantitative intensities, the effect of auto-activity for each bait protein can be systematically identified and normalized. For each bait X_i, background autoactivity level β_i was defined as ρ percentile of positive values amongst $s_{i,j}$ – med(s_i) where med(s_i) is median of $s_{i,j}$. Interaction signal *s'* was defined by ($s_{i,j}$ – med(s_i)) / β_i or 1 where $s_{i,j}$ – med(s_i) < β_i.For each protein pair, multiple *s'* signals are obtained from combinations of screening replicates (variants), pairs of differently assigned barcodes and the two barcode fusion types (BC1-BC1 and BC2-BC2 fusions). In order to find the best *s'* signal assembly method for each dataset, different ρ parameters are evaluated where each of Nth-ranking *s'* signals and average *s'* signal is defined as final interaction score to evaluate each protein pair.

The best scoring system, which has the best agreement with the previously reported Y2H dataset, is chosen based on the maximum of Matthews Correlation Coefficient (MCC_{max}).In the CENT screen, a total of 32 *s'* signal measurements were produced for each protein pair: two fused-barcode variants (BC1-BC1 and BC2-BC2) × four diploid replicates × two selective conditions × two library variants (with and without the seven auto-activators). The optimal MCC_{max} of 0.52 was achieved where ρ = 60% and 5th–ranking *s'* signals of the –His screens were adopted (**Appendix Figure S2**). The CCC screen was performed in two replicates without removal of auto-activating baits using two differently barcoded strains for each ORF (four each for the calibration set ORFs). The optimal MCC_{max} of 0.29 was achieved where ρ = 65% and 4th–ranking *s'* signals of the –His screens were adopted. Analysis scripts are available on request.

The *en masse* recombinational cloning-based BFG-Y2H screens

For each *en masse* Gateway-based BFG-Y2H screen, the data analysis to obtain interaction score matrix was performed as described above with the following exceptions: BC1s and BC2s for which total row sum or column sum counts in a +His screening matrix fell below a threshold γ were eliminated from the calculation; and for each of BC1-BC1 and BC2-BC2 fusion matrices, prey barcodes for which total column sum abundances were within the top τ were used to estimate β for each bait row. Step size for changing each of ρ, γ and τ parameters was arbitrarily defined, and for every ρ-γ-τ combination, 1st–ranking through 10th–ranking *s'* signals were calculated. Note that *s'* = 1 was adopted where *s'* of a given rank could not be calculated because of limited numbers of barcodes related to ORFs. The screening matrix size depended on the parameter τ. For each ρ-γ-τ-[*s'* rank] parameter combination, MCC_{max} for agreement with the previously-reported Y2H datasets was calculated to obtain interaction hits. Different CV and CVA interactome datasets were generated with different parameter combinations. Amongst those that yielded MCC_{max} >0.30, the ones which had the best agreement between CV and CVA were selected as the final interactome datasets. For CV, 389 interactions were obtained from a screening space size of 334,662 ORF pairs with the parameters ρ = 75%, γ = 400, τ = 1,000 and rank 2nd *s'*. For huCVA, 591 interactions were obtained from a screening space size of 2,574,869 protein pairs with the parameters ρ = 96%, γ = 500, τ = 100 and rank 3rd *s'*. Analysis scripts are available on request.

References

Bickle MB, Dusserre E, Moncorge O, Bottin H, Colas P (2006) Selection and characterization of large collections of peptide aptamers through optimized yeast two-hybrid procedures. *Nat Protoc* 1: 1066-1091

Neumann P, Navratilova A, Schroeder-Reiter E, Koblizkova A, Steinbauerova V, Chocholova E, Novak P, Wanner G, Macas J (2012) Stretching the rules: monocentric chromosomes with multiple centromere domains. *PLoS Genet* 8: e1002777

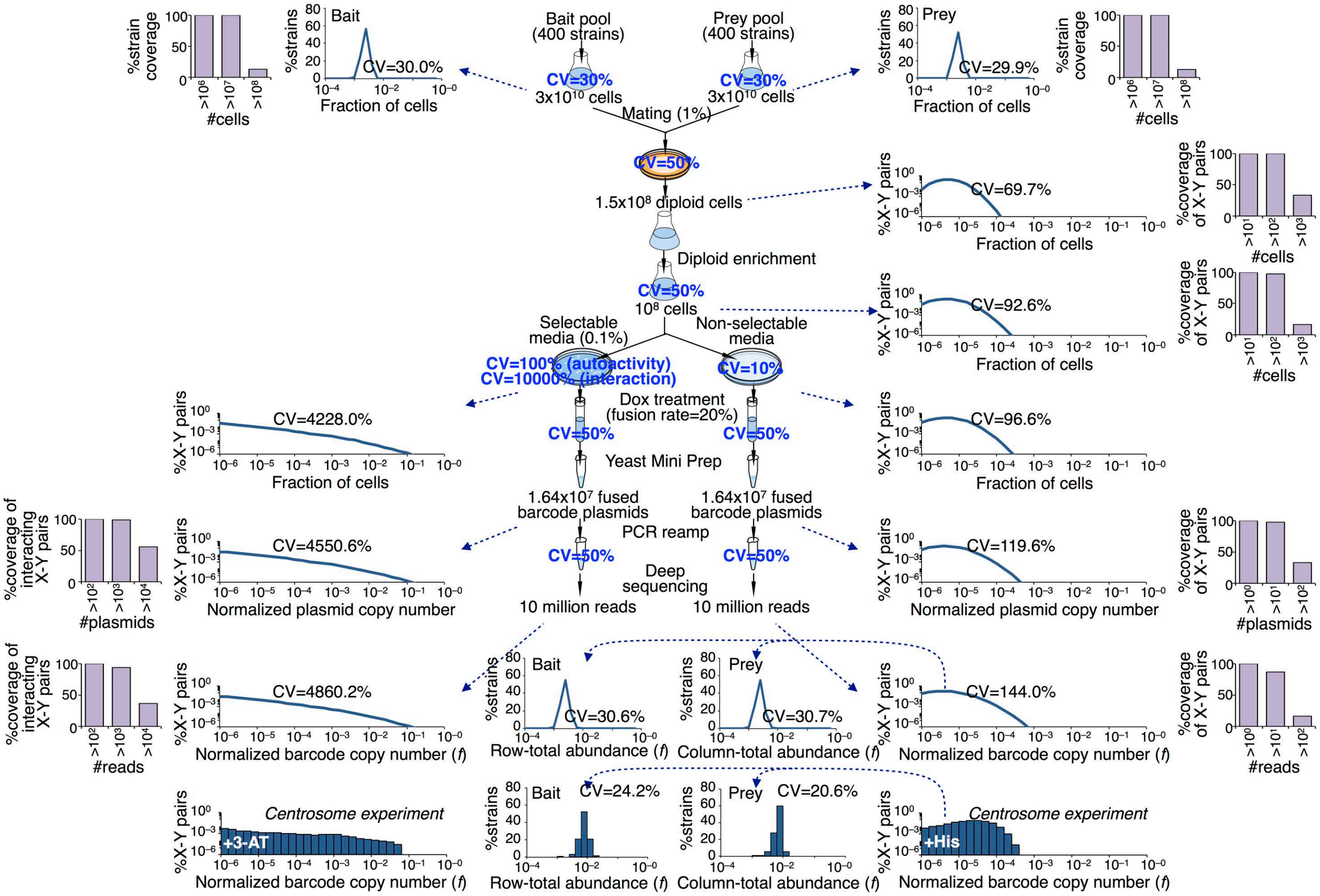

Appendix Figure S1. Monte-Carlo simulation of the BFG-Y2H process. See **Appendix Note S3** for details.

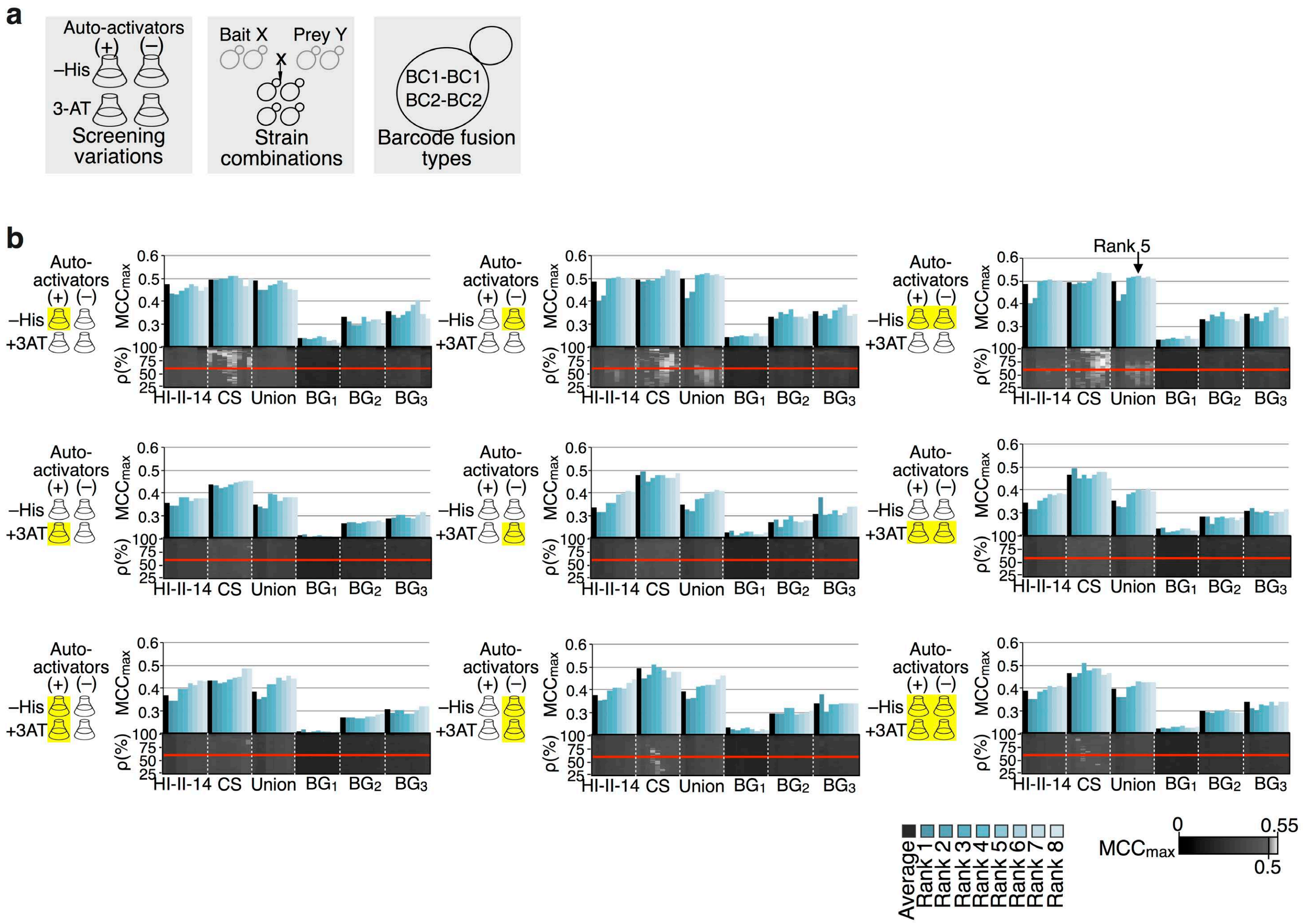

Appendix Figure S2. Performance of the CENT screen with various interaction scoring methods. (**a**) Multiple *s'* signals were obtained for each protein pair from the CENT screen, one for each of four screening variants (±Autoactivators × ±3-AT), four strain combinations (2 × 2 unique pairs) and two barcode fusion types (BC1-BC1 and BC2-BC2 fusions). (**b**) Performance of interaction scoring using different parameters and *s'* scoring methods. For each of the nine different screening variants, 1st-ranking *s'* through 8th-ranking *s'* signals and average *s'* signal, each derived using different ρ percentile parameters, were used to score interactions. Performance in terms of ability to recapture different categories of known interactions (six categories: HI-II-14, CS, Union, BG_1, BG_2 and BG_3) was measured by MCC_{max}. 'Union' denotes the union of interacting protein pairs in HI-II-14 and CS. BG_1, BG_2 and BG_3 are interaction datasets from the BioGRID database supported by one, two and three different interaction assay methods, respectively. Bar charts represent those of ρ = 60%. The yellow boxes indicate the scoring datasets adopted in each calculation.

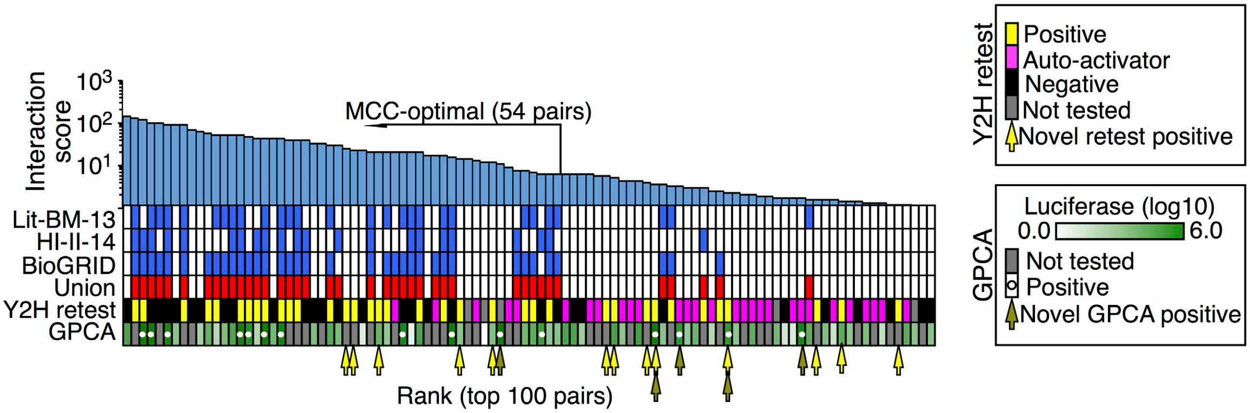

Appendix Figure S3. Top 100 protein pairs scored in the CCC screen. See Figure 5 for details.

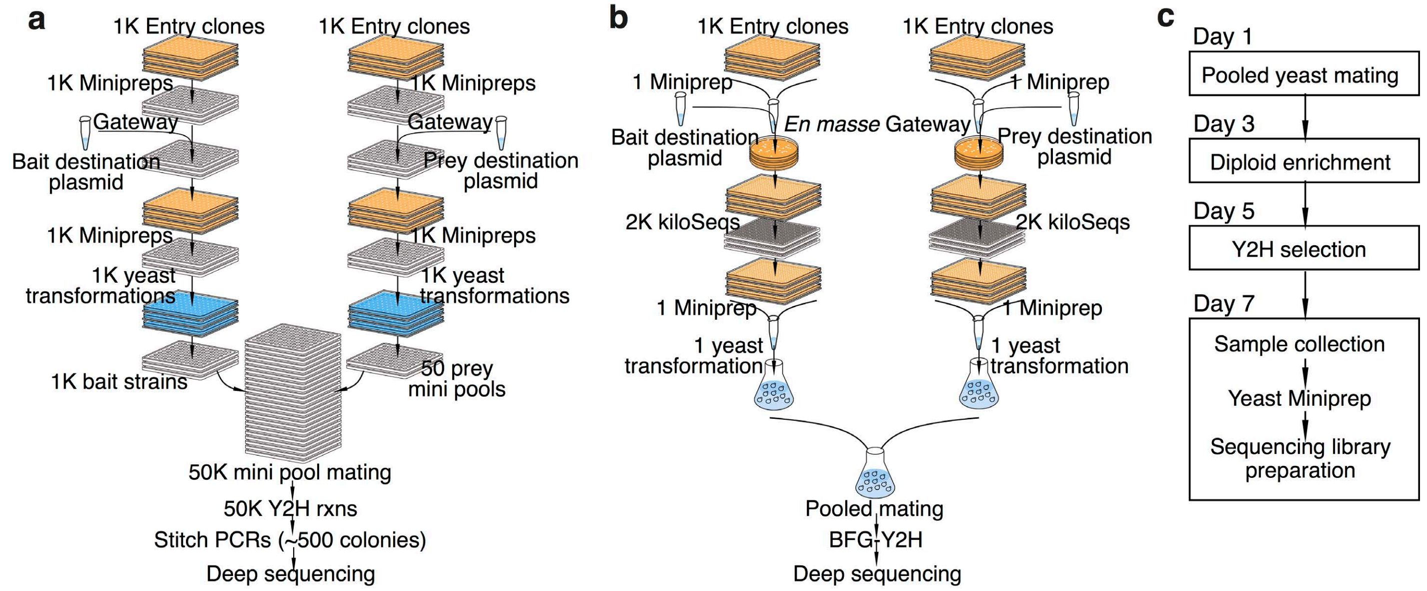

d

Kit/Reagent	Procedure	Unit price (USD/96-well plate)	Price (USD)
Miniprep kit	Miniprep 1K Entry clones (bait)	100	1,000
	Miniprep 1K Entry clones (prey)		1,000
	Miniprep 1K Expression clones (bait)		1,000
	Miniprep 1K Expression clones (prey)		1,000
LR Clonase	1K LR rxns (bait)	120	1,200
	1K LR rxns (prey)		1,200
Phusion polymerase	3 PCR rxns for each of 500 colonies	10	150
Deep sequencing	1 NextSeq500 run (High Output)	3,000	3,000

e

Kit/Reagent	Procedure	Unit price (USD/384-well plate)	Price (USD)
LR Clonase	EMRC (bait)	50	50
	EMRC (prey)		50
Phusion polymerase	kiloSeq 2K rxns (bait)	40	200
	kiloSeq 2K rxns (prey)		200
kiloSeq	kiloSeq 2K rxns (bait)	20	100
	kiloSeq 2K rxns (prey)		100
Deep sequencing	1 NextSeq500 run (High Output)	3,000	3,000

Appendix Figure S4. Cost comparison of primary screening by state-of-the-art Y2H versus *en masse* recombinational cloning (EMRC)-based BFG-Y2H. (**a**) Schematic diagram for primary screening using state-of-the-art Y2H with the Stitch-seq method to screen protein interactions from 1,000 baits × 1,000 preys of a given space. Vectors carrying ORFs of 1,000 bait and 1,000 prey entry plasmids are individually purified and subjected to one-by-one Gateway LR reactions. After the Gateway LR procedure, expression plasmids are purified from bacteria and individually transformed into Y8930 and 8800 strains, respectively. Prey mini-pools of 188 strains are generated, mated with each bait strain separately and subjected to Y2H selection. A total of 500 Y2H positive colonies are isolated. For each colony, the corresponding X-Y ORF pair is identified by the Stitch-seq pipeline, requiring three individual PCR reactions for each colony. (The number of colonies picked was estimated from the average number of colonies picked for the latest CCSB HI-II-14 screen.) (**b**) Schematic diagram of the EMRC-based BFG-Y2H pipeline for a given space of 1,000 baits and 1,000 preys. Bacterial cells of bait or prey ORF entry plasmids are all pooled and subjected to an *en masse* Gateway LR reaction with a pool of randomly barcoded bait or prey plasmids. Transformant colonies are isolated and the barcode sequence and ORF of each Expression plasmid is identified by kiloSeq. Clones bearing uniquely barcoded ORFs are rearrayed, pooled and used to transform RY1030 (for bait) and RY1010 (for prey) strains. BFG-Y2H is performed using the barcoded bait and prey strain pools generated. (**c**) Upon the strain preparation, BFG-Y2H requires 4 hands-on days and 7 total-screening days. (**d**) Kit/reagent costs estimated for the primary screening of 1,000×1,000 protein pairs by the state-of-the-art Y2H. (**e**) Kit/reagent costs estimated for the 1,000×1,000 screen by EMRC-based BFG-Y2H. BFG-Y2H is estimated to be 2.5-fold more economical than state-of-the-art Y2H screening. This estimate is conservative, as the cost reduction would be greater if the cost of plasticware and tips were included.
