# Supplementary material for: Pooled‐matrix protein interaction screens using Barcode Fusion Genetics
Source: Mol Syst Biol. 2016 Apr 23;12(4):863. doi: 10.15252/msb.20156660 (PMC4848762; doi:10.15252/msb.20156660)
Supplement: Supplementary file 2 — Expanded View Figures PDF [file MSB-12-863-s002.pdf]

Expanded View Figures

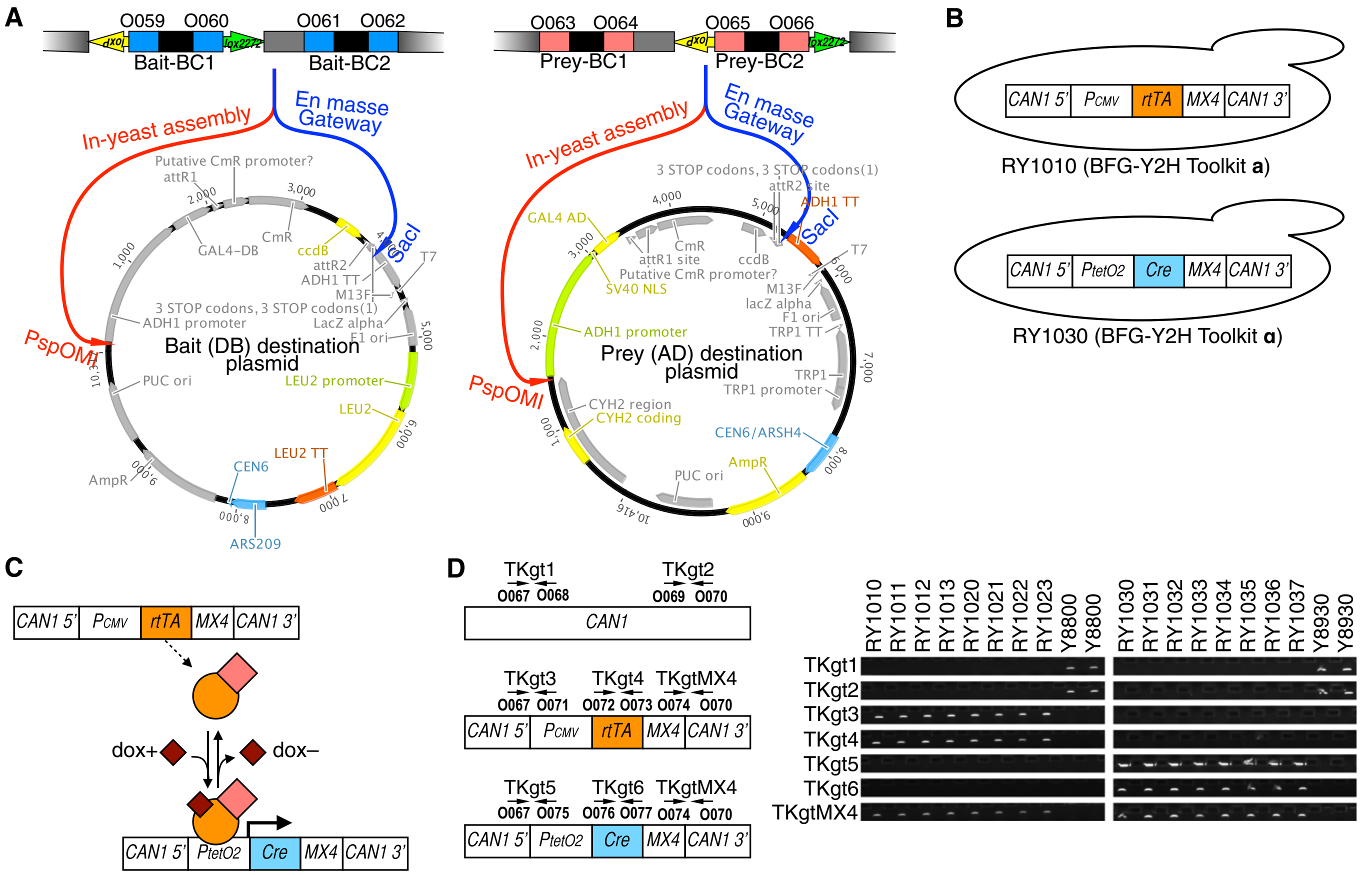

Figure EV1. Design of BFG-Y2H plasmids and strains.

A Design of barcoded bait and prey destination plasmids. Each destination plasmid carries two DNA barcodes (BC1 and BC2) interdigitated with *loxP* and *lox2272* sites. The barcoded bait or prey destination vectors harbor *loxP*′-BC1-*lox2272*-linker-BC2 or BC1-linker-*loxP*′-BC2-*lox2272* fragments at the PspOMI restriction site for the in-yeast assembly-based BFG-Y2H (red arrow), and at the SacI restriction site for the *en masse* recombinational cloning-based BFG-Y2H (blue arrow) so as to allow identification of barcode–ORF combinations in a paired-end sequencing read (*loxP*′ denotes the reverse complement of *loxP*). Each of the BC1 and BC2 regions is composed of a unique 25-bp DNA barcode flanked by common forward and reverse priming sites to allow barcode amplification by PCR: O059 and O060 sites for bait-BC1; O061 and O062 sites for bait-BC2; O063 and O064 sites for prey-BC1; and O065 and O066 PCR priming sites for prey-BC2.

B BFG-Y2H toolkit strains RY1010 (MAT**a**) and RY1030 (MATα) were constructed in a way that Cre recombinase expression can be induced only within diploids obtained by mating the two toolkit strains. A P_{CMV}-*rtTA-KanMX4* (toolkit-**a** cassette) fragment replaced the *CAN1* region of Y8800 chromosome and a T_{ADH1}-P_{tetO2}-*Cre*-T_{CYC1}-*KanMX4* (toolkit-α cassette) replaced the *CAN1* region of Y8930.

C Illustration of Tet-On system-based Cre expression. In the presence of doxycycline (dox), rtTA protein is activated, producing Cre via the *tetO*$_2$ promoter.

D Genotyping PCRs to confirm the toolkit strains. Successful creation of the toolkit strains was confirmed by direct PCRs for the strains RY1010, RY1030, Y8800, and Y8930. For each strain, the existence of the 5′ and 3′ regions of the wild-type *CAN1* was checked with O067 and O068 primers (TKgt1 PCR), and O069 and O070 primers (TKgt2 PCR), respectively. The integration of the toolkit-**a** cassette at the *CAN1* locus was checked by TKgt3 (amplification of the *CAN1 5*′/P_{CMV} boundary by O067 and O071 primers), TKgt4 (the *rtTA*-encoding region by O072 and O073 primers), and TKgtMX4 PCRs (the *MX4/CAN1 3*′ boundary by O074 and O070 primers). Finally, the integration of the toolkit-α cassette was checked by TKgt5 (the *CAN1 5*′/T_{ADH1} boundary by O067 and O075 primers), TKgt6 (the *Cre*-encoding region by O076 and O077 primers) and TKgtMX4 PCRs. All primer sequences can be found in Table EV5.

 © 2016 The Authors

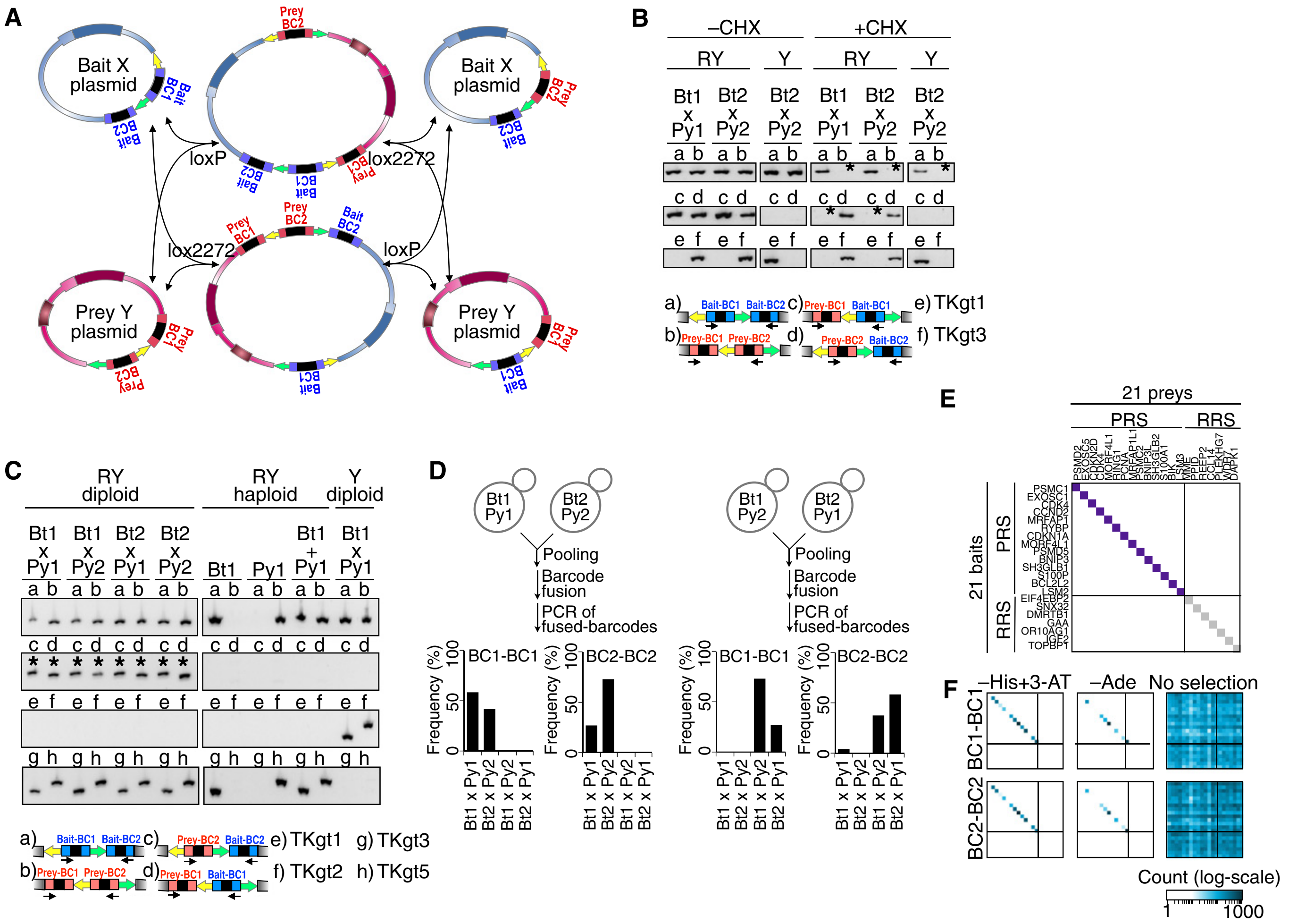

Figure EV2. Proof-of-principle demonstrations of BFG-Y2H.

A Major and shortest recombination pathways by which bait-BC1 and prey-BC2 are physically swapped between bait and prey plasmids.

B Barcode swapping on bait plasmid demonstrated by CHX treatment of various diploid strains to counter-select CYH2-encoding plasmids (see Fig EV1 for detail on the TKgt1 and TKgt3 genotyping PCRs). Evidence of loss of plasmid due to CHX treatment is indicated by "*". "RY" represents our toolkit strain background, and "Y" represents the Y-strain background commonly used in latest Y2H experiments (Appendix Note S1).

C Genotyping PCR of fused and unfused barcodes in various strain backgrounds (see Fig EV1 for description of TKgt1/2/3/5 PCRs). A PCR product corresponding to a fused barcode is indicated by "*".

D Frequencies of BC1-BC1 and BC2-BC2 fused barcodes obtained from a mixture of two diploid strains, such that each diploid strain harbored uniquely barcoded bait and prey plasmids.

E, F Demonstration of small-scale BFG-Y2H. (E) 14 positive reference set (PRS) pairs (navy cells) and 7 random reference set (RRS) pairs (gray cells) were chosen from the CCSB human PRS and RRS version 1 (hsPRSRRSv1). The 14 PRS pairs were reported to be Y2H positive in both of the X-Y and Y-X configurations by pairwise testing. (F) BFG-Y2H screens for the small-scale matrix with –His+3-AT, –Ade, and no-selection control (+His +Ade) conditions.

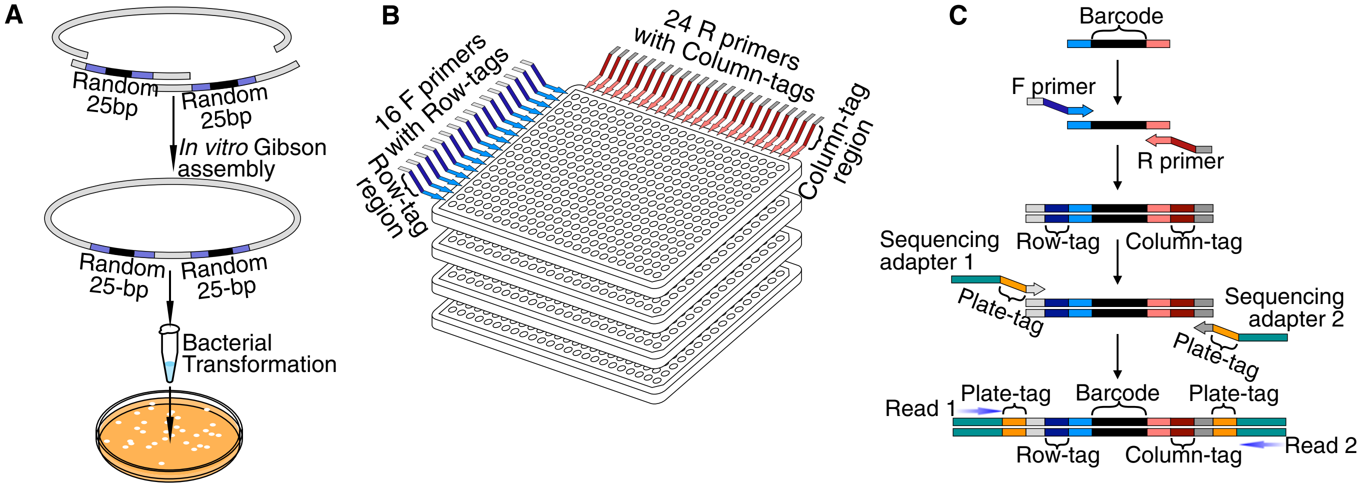

Figure EV3. Rapid generation of DNA barcode collections.

A Two oligonucleotide DNA pools harboring random 25-bp sequence regions were combined with site-specific recombination sites (*loxP* and *lox2272*) by PCR, assembled with a linear plasmid backbone fragment by Gibson assembly. The resulting randomly barcoded plasmid pool was transformed to *E. coli* cells.

B Single randomly barcoded *E. coli* colonies were picked and isolated into 384-well plates, and the pair of unique barcodes in each well was identified by row–column plate (RCP)-PCR (Appendix Note S2). RCP-PCR was designed to determine the identity and plate and well location of barcode sequences in many strains arrayed in microwell plates via a single next-generation sequencing run. In a 384-well format reaction, 16 forward primers with row-specific DNA index tags and 24 reverse primers with column-specific DNA index tags are distributed to their corresponding row and column positions. The forward and reverse row-/column-specific primers also have plate primer landing sites on their ends.

C RC-PCRs are performed for individual plates to stitch row and column tags to each barcode. RC-PCR products are then pooled by plates. A Plate-PCR stitches plate index tags and Illumina paired-end sequencing adapters to each RC-PCR product pool. The plate-PCR products are pooled and sequenced *en masse* to identify the sequence of barcode regions at every row–column plate coordinate.

© 2016 The Authors

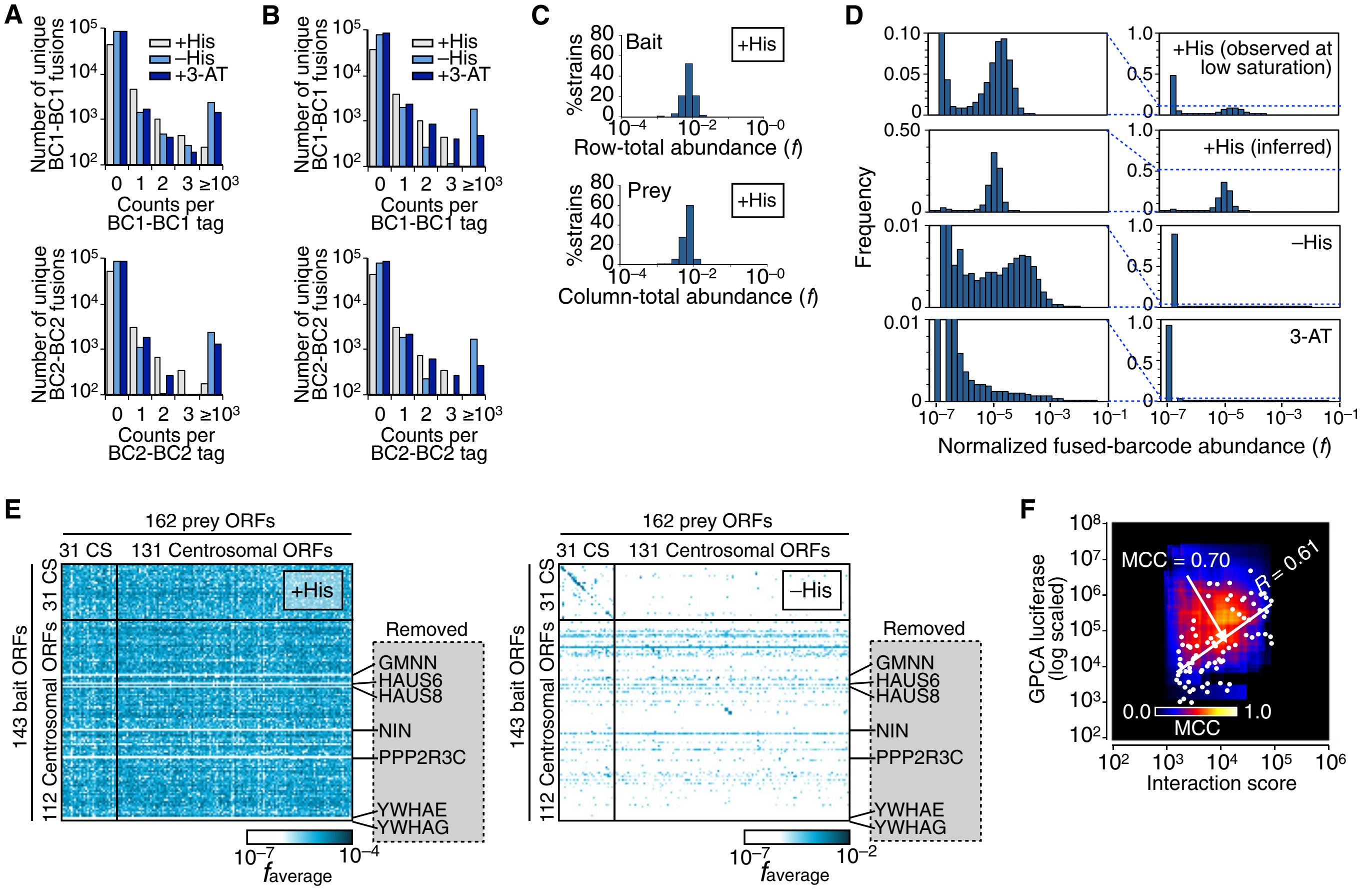

Figure EV4. Additional information on the CENT screen.

A, B Distribution of fused-barcode counts in the non-selective (+His), selective (–His), and stringent selective (3-AT) conditions with (A) and without (B) the seven auto-activators.

C Distributions of normalized row-total abundances and column-total abundances in the non-selective condition (+His, with auto-activators), inferred distributions of pre-mating bait and prey haploid strain abundance, respectively.

D, E Information on the CENT screen performed without the seven auto-activators. (D) Distribution of normalized fused-barcode abundance observed in non-selective conditions (+His observed at low saturation), inferred for the non-selective condition using row- and column-total abundances (+His inferred) observed in the selective conditions (–His and 3-AT). (E) Average of normalized fused-barcode count for each ORF pair ($f_{average}$) in the non-selective (+His) condition and the selective (–His) condition. CS: calibration set space spiked in the screen.

F Correlation between BFG-Y2H interaction scores and GPCA luciferase intensities. The background MCC heat map demonstrates overlap of the two datasets at each threshold combination.

© 2016 The Authors

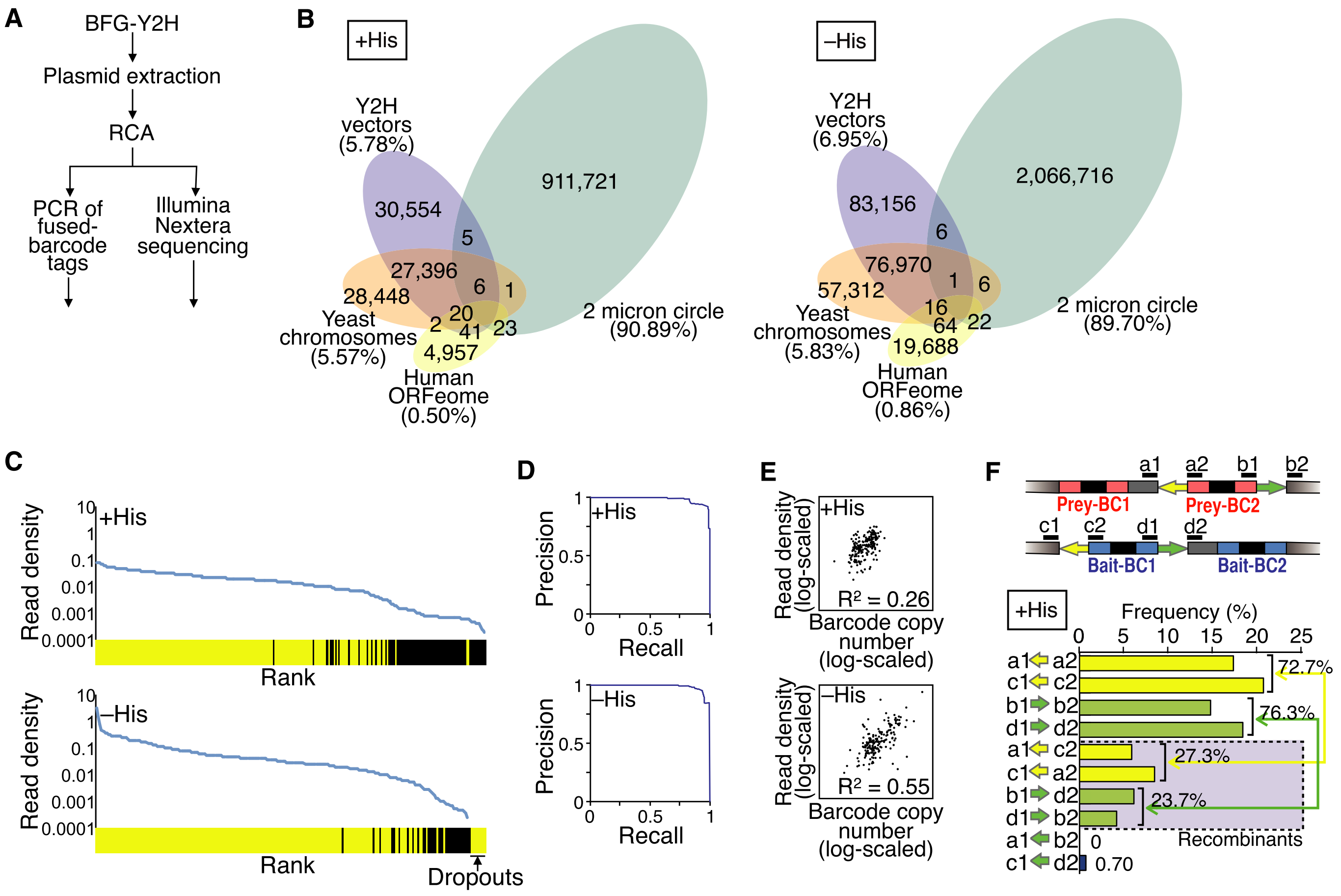

Figure EV5. Sequencing of extracted plasmid pools after induction of barcode fusion.

A Schematic diagram of the experimental flow. After the yeast plasmid DNA extraction during the CENT screen, in a separate experimental procedure, plasmid DNA samples of +His and –His conditions were amplified by φ29 polymerase-based rolling circle amplification (RCA). Fused barcodes were then amplified by PCR from the RCA-treated DNA pool and sequenced (Illumina MiSeq). In parallel, entire-plasmid DNA pools were sequenced (Illumina Nextera library preparation sequenced on an Illumina MiSeq).

B Counts of Nextera sequencing reads mapped using appropriate reference databases.

C Human ORFs found among entire-plasmid sequencing reads. ORFs were sorted according to the read density, defined as their read counts divided by ORF length (reads/bp). Yellow bars denote centrosomal ORFs interrogated in the screen; and black bars denote "unexpected" ORFs. The sequencing result of the +His condition covered all of the centrosomal ORFs, while some centrosomal ORFs were not found in the –His Y2H screening condition (dropouts), as expected given that not all ORFs encode interacting proteins.

D Precision-recall curve assessing separation of centrosomal ORFs from non-centrosomal ORFs by read density among ORFs found by Nextera sequencing.

E Correlation between ORF read densities and corresponding barcode copy numbers.

F Estimation of barcode fusion efficiency from heptamer–*lox*–heptamer combinations found in the Nextera sequencing reads of the +His condition (the –His condition data can be found in Fig 4E). Yellow and Green arrows denote *loxP* and *lox2272* sites, respectively.

 © 2016 The Authors
